# Afterimage watercolors: an exploration of contour-based afterimage filling-in

**DOI:** 10.3389/fpsyg.2013.00707

**Published:** 2013-10-08

**Authors:** Simon J. Hazenberg, Rob van Lier

**Affiliations:** Donders Institute for Brain, Cognition and Behaviour, Radboud University NijmegenNijmegen, Netherlands

**Keywords:** color, contours, afterimages, filling-in, illusion

## Abstract

We investigated filling-in of colored afterimages and compared them with filling-in of “real” colors in the watercolor illusion. We used shapes comprising two thin adjacent undulating outlines of which the inner or the outer outline was chromatic, while the other was achromatic. The outlines could be presented simultaneously, inducing the original watercolor effect, or in an alternating fashion, inducing colored afterimages of the chromatic outlines. In Experiment 1, using only alternating outlines, these afterimages triggered filling-in, revealing an “afterimage watercolor” effect. Depending on whether the inner or the outer outline was chromatic, filling-in of a complementary or a similarly colored afterimage was perceived. In Experiment 2, simultaneous and alternating presentations were compared. Additionally, gray and black achromatic contours were tested, having an increased luminance contrast with the background for the black contours. Compared to “real” color filling-in, afterimage filling-in was more easily affected by different luminance settings. More in particular, afterimage filling-in was diminished when high-contrast contours were used. In the discussion we use additional demonstrations in which we further explore the “watercolor afterimage.” All in all, comparisons between both types of illusions show similarities and differences with regard to color filling-in. Caution, however, is warranted in attributing these effects to different underlying processing differences.

## Introduction

Perception of color does not always reflect what is physically present. This is clearly demonstrated in the case of colored afterimages where, after adapting to a colored stimulus, a vivid afterimage of a complementary hue is seen when the stimulus is removed or if one changes their gaze to a blank wall. Although the neural locus of the afterimages is still debated, data from a recent study indicates that the appearance of colored afterimages arise from signals originating in the retina (Zaidi et al., 2012). It has been argued further that the signals coding for colored afterimages may be, like retinal signals coding for “real” colors, subject to various kinds of contextual modifications. In this study we focused on the influence of luminance contours on the perception of colors. Specifically, we investigated filling-in of colored afterimages between luminance contours and compared this with color filling-in that is induced by “real” colors in the well-known watercolor illusion.

**Figure 1:**
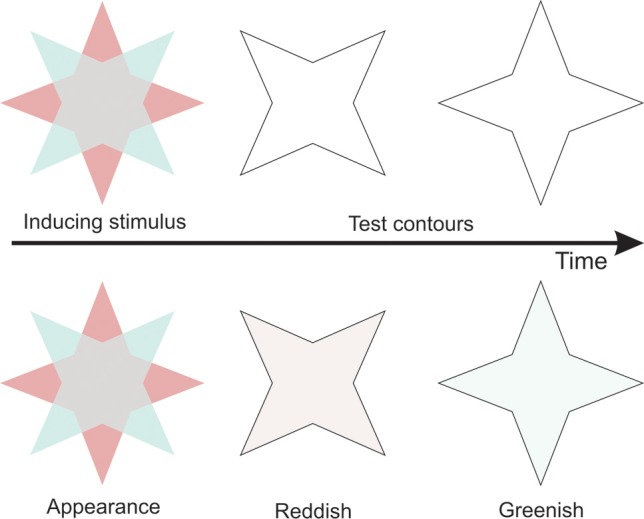
**Illustration of the stimuli used in van Lier et al. (2009)**. The first row shows the stimuli. The second row shows the resulting percepts.

There have been various studies showing that the perception of colored afterimages can be modulated by luminance defined contours. For instance, an afterimage appears much more salient and saturated when it is surrounded by a luminance contour (Daw, 1962). More recently, the dependence of afterimages on contours have been emphasized by showing that a colored afterimage spreads within a test outline and fills in regions that were not adapted to color (van Lier et al., 2009). They used adapting stimuli consisting of multiple colors and an achromatic inner region and showed that the location that was not adapted to color revealed an afterimage color that depended on the subsequent presented outline (see Figure 1). They additionally demonstrated that when afterimages of multiple colors are contained within a contour, the colors within that contour tend to mix (van Lier et al., 2009; Anstis et al., 2012a). The color of an afterimage not only depended on the color that was positioned inside a subsequent contour, but also on the color that was positioned outside the subsequent contour. The color inside the contour produced a complementary color, whereas the color outside the contour produced a color that was similar to the inducing color, although the latter effect was found to be less strong (van Lier et al., 2009). Two possible routes have been suggested previously to explain the latter colored afterimage. Along the first route, the outside color induces a contrasting color across the boundary into the interior of the figure during the adapting period which then produces a complementary colored afterimage during the test phase. Along the second route, the outside color produces a complementary colored afterimage during the test phase, which then induces a contrasting color into the interior of the outline (Anstis et al., 1978).

**Figure 2:**
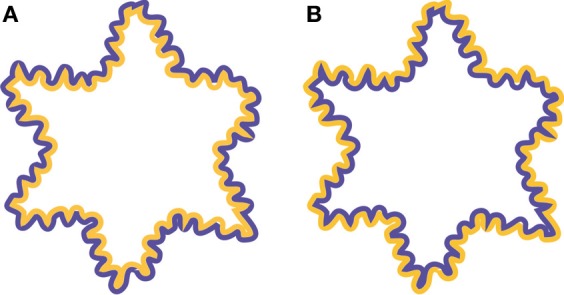
**Examples of the watercolor illusion. (A)** By juxtaposing the lighter colored contour (orange) to a darker colored contour (purple) inside an enclosed area, color appears to spread inwards and uniformly fills the interior of the star-like shape. **(B)** Reversing the colors of the contour results in color spreading outwards.

Interactions between contours and “real” colors are known as well. Chromatic sensitivity seems to be enhanced when a colored region is bound by luminance edges (Montag, 1997). Similarly, “real” color filling-in occurs in the Boynton illusion in which color spreads out from a colored region to (nearly) isoluminant achromatic areas until it reaches luminance-defined contours or illusory contours (Feitosa-Santana et al., 2011). Furthermore, in the neon color illusion color diffuses from colored parts of, for example, multiple concentric lines and is blocked by illusory contours (van Tuijl, 1975; Bressan et al., 1997; van Lier, 2002). The typical percept is of an illusory colored transparent disc floating in front of the inducing elements. Finally, Pinna et al. (2001) developed a clear demonstration that even a pair of juxtaposed thin colored undulating outlines induces color filling-in, i.e., the so-called watercolor illusion. Figure 2A shows a typical example of the watercolor illusion. By positioning the light orange outline on the inside and the dark purple outline on the outside of the star-like shape, the interior of the shape appears to be filled-in with a color that is similar, but less saturated than the color of the inner contour. When the colors of the outlines are reversed, color appears to spread outwards (Figure 2B). Color then, spreads in the direction where the luminance contrast between the contour and the background is lowest. In addition, the strength of the effect depends on luminance contrast between the two outlines. When the outlines are isoluminant, color spreading is rather weak and appears to spread both inwards and outwards, but becomes more vivid when the luminance contrast between the outlines is enhanced (Devinck et al., 2005). Additionally, Cao et al. (2011) showed that there is an optimal contrast after which color spreading diminishes again.

In any case, it seems that the appearance of surface color strongly depends on edge information. This is in line with a study in which the activity of single neurons in V1 and V2 were recorded (Friedman et al., 2003). They showed that some cells that code for color are also orientation selective, indicating that the representation for form and color are tightly linked (Von der Heydt and Pierson, 2006). Activity in these cells might be responsible for filling-in of both afterimage colors and “real” colors. Similarly, activity of cells with receptive fields along the edges might also underlie perceived contrast induction of colors across edges. Indeed, in a version of the watercolor illusion in which the inner contour of a shape was achromatic (black) and the outside contour was colored, a complementary color was perceived in the interior of the shape (Pinna, 2006).

Given the similarity of the observed interactions between colors and luminance contours in both “real” colors and afterimage colors it seems plausible that the observed effects tap from a common mechanism. In this study, we further explored this by comparing performance on two color judging experiments in which filling-in could be induced by afterimage colors or by “real” colors. Similar to the stimuli used to investigate the watercolor illusion, we only used thin outlines. In Experiment 1, we first investigated whether the afterimage filling-in effects described by van Lier et al. (2009) also occur when using thin colored outlines similar to the watercolor illusion. In Experiment 2, we used such stimulus configurations to compare filling-in of afterimage colors with filling-in in the watercolor illusion.

## Experiment 1

In this experiment, a chromatic contour alternated over time with an achromatic contour. Thus, when the achromatic contour is presented, an afterimage of the previously presented chromatic contour should be perceived. The chromatic contour could be positioned inside or outside a subsequently presented achromatic contour. Participants had to judge the color of the interior of the achromatic outline. If afterimage colors of thin outlines are sufficient to induce color filling-in, the interior of the figures should reveal complementary color filling-in when the chromatic outline is placed inside the achromatic outline, whereas filling-in of a similar color as the chromatic outline should be induced when this outline is positioned outside the achromatic outline. Following van Lier et al. (2009) we further expected to find weaker color filling-in for outer chromatic contours as compared to inner chromatic contours.

### Methods

#### Participants

Twenty-one observers participated in Experiment 1 (aged 17–24, one participant had an age of 64; six males). All had normal or corrected to normal visual acuity. In addition, all participants had normal color vision as screened with the AO Hardy Rand and Rittler Pseudoisochromatic Plates (2nd edition). Participants received payment or course credits. All participants were naive to the purpose of the experiment.

#### Stimuli

**Figure 3:**
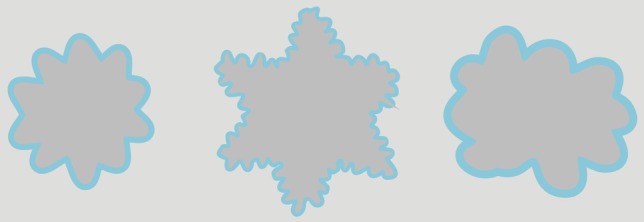
**Examples of adapting stimuli**.

Stimuli were presented on a gray background [*CIE*_(*x, y*)_ = 0.3128, 0.3303] with a luminance of 73.87 cd/m^2^. The stimuli consisted of closed, arbitrarily undulating, contours. Three outlines or contours were created that differed in shape, size, and thickness (see Figure 3 for examples of the contours). A second set of contours was created such the shapes of these contours fit neatly within the shapes of the former set of contours. Thus, for each shape in Figure 3, we distinguished between an “outer” contour and an “inner” contour.

Both inner and outer contours could be used as adapting stimuli or as test stimuli. The contours of the chromatic adapting stimuli could be colored either green [*CIE*_(*x,y*)_ = 0.3514, 0.4417; *L* = 65.69 cd/m^2^], orange [*CIE*_(*x,y*)_ = 0.3774, 0.3694; *L* = 58.65 cd/m^2^], blue [*CIE*_(*x,y*)_ = 0.2600, 0.3058; *L* = 52.90 cd/m^2^], or pink [*CIE*_(*x,y*)_ = 0.3814, 0.2733; *L* = 29.87 cd/m^2^]. The colors were chosen such that two colors were approximately complementary to each other (e.g., orange-blue; pink-green). The interior of the adapting contour was gray having a luminance that was equal to the luminance of that contour. This was done as luminance borders between the inducing color and the interior area may block afterimage filling-in (van Lier et al., 2009). The contours of the achromatic test stimuli were gray and had a luminance of 55 cd/m^2^. Note that all stimuli were darker than the background. Examples of stimuli in which the inner contour is the adapting chromatic contour are shown in Movie 1 and examples of stimuli in which the outer contour is the adapting chromatic contour are shown in Movie 2.

#### Procedure

The experiment was run on a PC and an 18-inch CRT monitor with a 120 Hz refresh rate. The monitor was calibrated using an X-Rite Color Monitor Optimizer. The experiment was designed and presented using Presentation (Version 14.8, Neurobehavioral Systems, Inc.).

**Figure 4:**
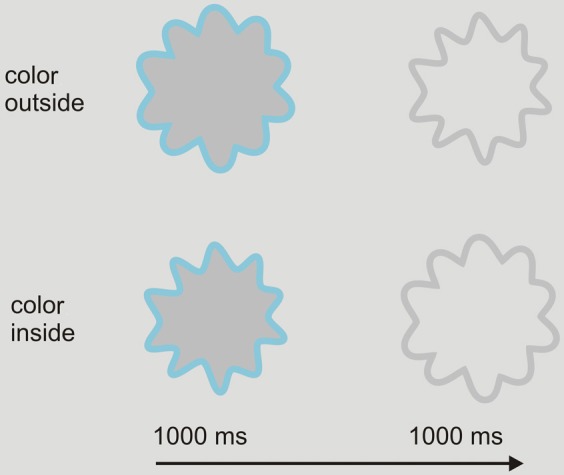
**Sequence of events in Experiment 1. Upper row:** an example of a trial in which the outer contour is the chromatic stimulus and the inner contour is the achromatic test contour. **Lower row:** an example of a trial in which the inner contour is the chromatic stimulus and the outer contour is the achromatic test contour.

Once written informed consent was given and the instructions were read, the experiment started. The sequence of events is shown in Figure 4. Each trial started with the presentation of a small fixation dot that was presented on the center of the screen for 1000 ms. Afterwards a chromatic adapting stimulus was presented for 1000 ms which was followed by an achromatic test stimulus that was presented for another 1000 ms. The adapting stimulus and test stimulus kept alternating until a response was made. Once a response was made, the next trial started automatically after 1000 ms.

We distinguished between two types of trials (See Figure 4). In the first type, the outer contour was the chromatic adapting stimulus and the inner contour was the achromatic test stimulus. In the second type, the inner contour was the chromatic adapting stimulus, while the outer contour was the achromatic test stimulus. Participants were instructed to judge whether the inside of the achromatic test stimulus appeared to be filled in with a color. In order to respond, five disks, four of which were colored, were shown at the bottom of the screen. The colors of the response disks were similar to the inducing colors. When participants perceived color filling-in, they had to choose which of the colored response disks best matched the perceived color. The fifth response disk comprised the same gray as the background and could be chosen whenever participants did not perceive any color filling-in. Participants were asked to observe at least three presentation cycles (chromatic contour, achromatic contour) before responding. Responses were given by pushing one of five buttons corresponding to the five disks.

There were 24 unique trials; stimulus shape (3 levels) × color (4 levels) × trial type (2 levels). Prior to the experiment, participants completed a small practice block consisting of three trials to get familiar with the task. The main experiment was administered in four blocks. In each block, each of the 24 trials was presented once in a randomized order. Participants controlled the time between the blocks and started the next block by pressing one of the five response buttons.

### Results

**Figure 5:**
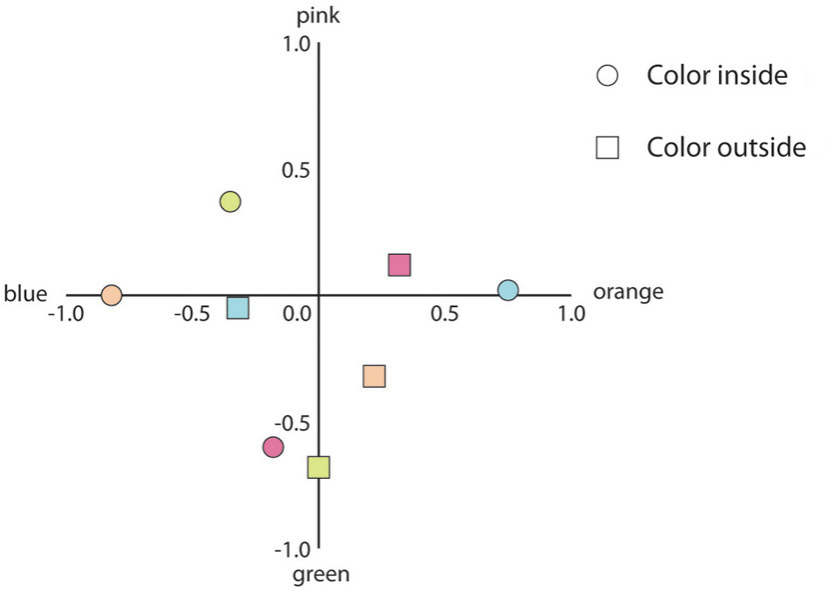
**Mean response coordinates when the inner or the outer contour was the adapting chromatic stimulus (indicated by circles and squares, respectively)**. The color of the symbols within the graph depict the colors of the adapting stimuli (e.g., the rightmost blue circle indicates the mean response for the adapting stimuli in which the blue contour was positioned inside the gray contour).

One participant was unable to perceive any afterimages and was not included in the analysis. In Figure 5 the responses are plotted. The responses for the colors orange, blue, pink and green were assigned to coordinates (1, 0); (–1, 0); (0, 1); (0, –1), respectively. For example, a 100% response for the color orange is represented by plot coordinate (1, 0). Additionally, a no color response was assigned to coordinates (0, 0). Figure 5 shows the coordinate plots for trials in which the inner contour was the chromatic contour and for trials in which the outer contour was the adapting contour.

To analyze the data, we calculated the proportions each participant responded with “same color,” “complementary color,” and “no color.” The resulting proportions were transformed using the arcsine transformation in order to obtain a normal distribution of the data. All statistical tests were performed on these transformed proportions. Paired *t*-tests showed that when the inner contour was chromatic, the proportion complementary color responses was larger as compared to the proportion same color responses [*t*_(19)_ = 11.98, *p* < 0.001]. In contrast, when the outer contour was chromatic, there were more same color responses as compared to complementary color responses [*t*_(19)_ = 5.18, *p* < 0.001]. We also compared the proportions no color responses to check whether the probability of perceiving any color filling-in differed between conditions. A paired *t*-test revealed significant results [*t*_(19)_ = 2.41, *p* < 0.05], showing that participants were more likely to respond with no color when the outer contour was chromatic as compared to when the inner contour was chromatic.

### Discussion

The results showed that afterimages of chromatic outlines filled in regions that were not adapted to color. The color of the filled-in region depended on the position of the chromatic contour in the adapting stimulus: when the chromatic contour was positioned at the inside, filling-in of the complementary color in the inner area was perceived; when the chromatic outline was positioned outside, filling-in of the same color in the inner area was perceived. Afterimage filling-in induced by the outer contour appeared less strong as compared to afterimage filling-in induced by the inner chromatic contour, although there appears to be some variability within the different colors (e.g., compare green inside and outside in Figure 5 with the other colors).

The fact that thin colored outlines were sufficient to induce spreading within closed boundaries strengthened our initial idea that the filling-in mechanisms triggered by afterimage colors proceeds in a similar fashion as the filling-in processes that are at work in the original watercolor illusion. The next experiment was set up to further test similarities between both types of filling-in.

## Experiment 2

We used a color judging experiment similar to Experiment 1. In addition to presenting the chromatic and achromatic contours alternately, watercolor-like stimuli were created by presenting the contours simultaneously. We expected filling-in of different colors to depend on whether the contours alternated or whether they were presented simultaneously. For example, an inner chromatic contour should induce color spreading of a similar hue when the contours are presented simultaneously, but the same chromatic contour should induce complementary color filling-in when the contours alternated. Previous studies found that the strength of the watercolor illusion depends on the asymmetric luminance profiles of both contours (Pinna et al., 2001; Devinck et al., 2005). To test the effect of luminance contrast between chromatic and achromatic outlines, we used two kinds of achromatic contours. One was of a same gray as in Experiment 1, while the other was much darker (i.e., black). We expected that when the contours were presented simultaneously, stronger color filling-in should be perceived when black achromatic contours were used as compared to gray achromatic contours. Note that in Experiment 1, the inner area was isoluminant to the chromatic contour to enhance filling in. However, to allow a better comparison with the usual static watercolor illusion, in the current experiment the inner area has the same luminance as the surrounding background, which is different from the luminance of the chromatic contour.

### Methods

#### Participants

Twenty observers participated in Experiment 2 (aged 17–24; four male). All had normal or corrected to normal visual acuity. In addition, all participants had normal color vision as screened with the AO Hardy Rand and Rittler Pseudoisochromatic Plates (2nd edition). Participants received payment or course credits. All participants were naive to the purpose of the experiment.

#### Stimuli

**Figure 6:**
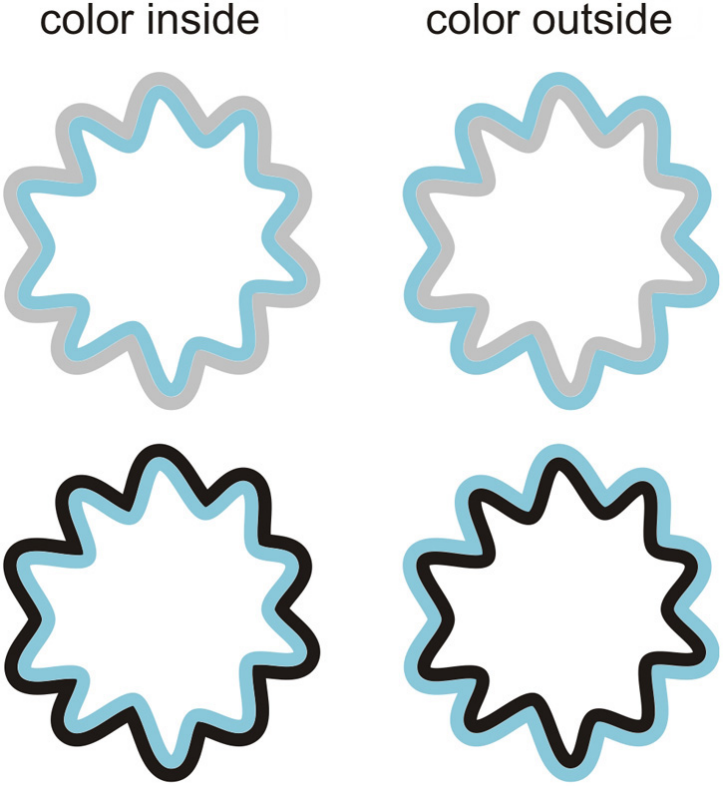
**Example stimuli that were used to induce the watercolor effect**. Both the inner contour (**left panels**) and the outer contour (**right panels**) could be chromatic. The achromatic contour could either be gray (**upper panels**) or black (**lower panels**).

The stimuli we used in this experiment were similar to those we used in Experiment 1, but with the following changes. Firstly, in addition to presenting the contours in an alternating fashion, the chromatic and achromatic contours where also presented simultaneously to induce the classic watercolor illusion. Examples of these stationary stimuli are shown in Figure 6. Secondly, the interior of the adapting contours was always of the same white as the background. In order to enhance the watercolor effect, stimuli were presented on a white background (100 cd/m^2^). Lastly, in addition to the gray contour that was used in Experiment 1, we also used black contours (0.39 cd/m^2^).

#### Procedure

For the alternating presentation condition, the same procedure as in Experiment 1 was followed. Similarly, in the simultaneous presentation condition, participants had to judge whether the interior of the stimulus appeared colored or not, using the same response categories as in Experiment 1. The stimuli remained on the screen until participants responded.

There were 96 unique trials: stimulus shape (3 levels) × color (4 levels) × trial type (2 levels; inner or outer contour was chromatic) × presentation type (2 levels; alternating or simultaneous) × achromatic contour (2 levels; black or gray). Each unique trial was presented twice. The experiment was administered in four blocks in which the variables presentation type and achromatic contour were blocked while the other trials were presented randomly. The presentation order of the blocks was counterbalanced across participants. In each block, each of the 24 trials was presented once in a randomized order. Participants controlled the time between the blocks and started the next block by pressing one of the five response buttons.

### Results

**Figure 7:**
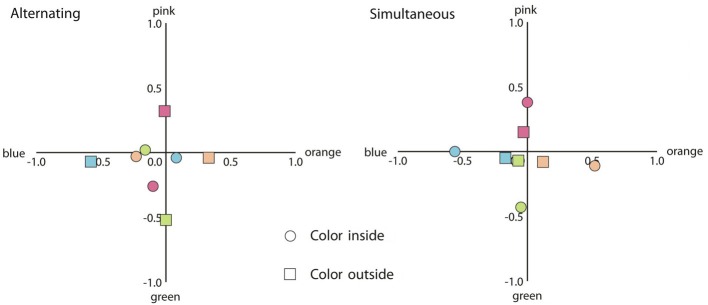
**Mean response coordinates when the inner or the outer contour was the adapting chromatic stimulus (indicated by circles and squares, respectively) and the achromatic contour was gray. Left panel:** mean responses for the alternating presentation condition. **Right panel**: mean responses in the simultaneous presentation condition. The color of the symbols within the graph depicts the colors of the adapting stimuli.

We have plotted the results in a similar fashion as the results of Experiment 1. First we consider the results of trials when the achromatic contours were gray (see Figure 7).

The results were analyzed according to the response categories specified in Experiment 1. As in Experiment 1, all analyzes were performed on the arcsine transformation of the proportions. As can be seen in Figure 7, for the alternating presentation condition, when the outer contour was chromatic, the proportion participants responded with the same color was greater as compared to the proportion participants responded with the complementary color [*t*_(19)_ = 4.13, *p* < 0.01]. However, when the inner contour was chromatic, the same color responses and the complementary color responses did not appear to differ (*p* > 0.1). For the simultaneous presentation condition, the same color response prevailed as compared to the complementary color response for both the inner and the outer chromatic contour [*t*_(19)_ = 7.80, *p* < 0.001; *t*_(19)_ = 3.53, *p* < 0.01; respectively]. To analyze whether the probability of perceiving any color filling-in differed between conditions, we also compared the no color responses. Paired *t*-tests showed no difference for the alternating presentation condition (*p* > 0.1). For the simultaneous presentation condition, participants were more likely to respond with no color when the outer contour was chromatic as compared to when the inner contour was chromatic [*t*_(19)_ = 3.89, *p* < 0.01].

**Figure 8:**
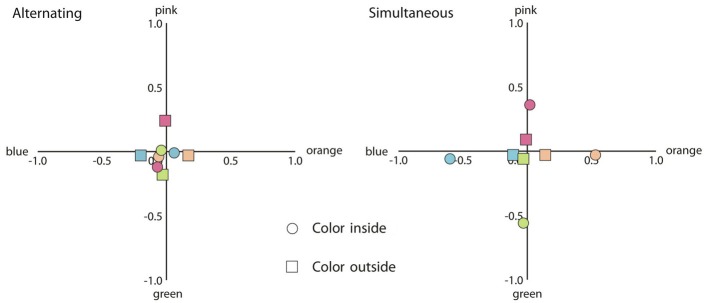
**Mean response coordinates when the inner or the outer contour was the adapting chromatic stimulus (indicated by circles and squares, respectively) and the achromatic contour was black. Left panel:** mean responses for the alternating presentation condition. **Right panel:** mean responses in the simultaneous presentation condition. The color of the symbols within the graph depicts the colors of the adapting stimuli.

Next we consider responses on the same conditions, but now for the stimuli with black achromatic contours (See Figure 8).

As can be seen in Figure 8, the same pattern as before was found. For the alternating presentation condition, when the outer contour was chromatic, the proportion participants responded with the same color was greater as compared to the proportion participants responded with the complementary color [*t*_(19)_ = 3.40, *p* < 0.01]. However, when the inner contour was chromatic, the same color responses and the complementary color responses did not differ (*p* > 0.1). For the simultaneous presentation condition, participants were more likely to respond with the same color response as compared to the complementary color response for both the inner and the outer chromatic contour [*t*_(19)_ = 8.80, *p* < 0.001; *t*_(19)_ = 2.48, *p* < 0.05; respectively]. Paired *t*-tests on the proportions participants responded with no color revealed no difference for the alternating presentation condition (*p* > 0.1). However, for the simultaneous presentation condition, participants were more likely to respond with no color when the outer contour was chromatic as compared to when the inner contour was chromatic [*t*_(19)_ = 3.43, *p* < 0.01].

For the alternating presentation condition, we noticed that when black achromatic contours were used, color filling-in appeared to be attenuated as compared to when the lighter gray achromatic contours were used. Paired *t*-tests confirmed this, showing that for both inner [*t*_(19)_ = 2.85, *p* < 0.05] and outer chromatic contour [*t*_(19)_ = 5.18, *p* < 0.001], the proportion participants responded with no color was greater when black contours were used as compared to when gray contours were used. No such effect was found for the simultaneous presentation condition.

### Discussion

In this experiment we compared filling-in of afterimage colors with filling-in of “real” colors. We used chromatic and achromatic contours that could be presented simultaneously or alternately. When gray achromatic contours were used, for the alternating condition, an outer chromatic contour induced filling-in of a similar color whereas an inner chromatic contour hardly induced filling-in of the expected complementary color. In contrast, for the simultaneous presentation condition, the probability of perceiving color filling-in was greater for an inner chromatic contour as compared to an outer contour. Furthermore, filling-in induced by an inner or an outer chromatic contour was most likely to be similar to the color of the contour. Although we expected to find stronger color spreading for the simultaneous presentation condition when black achromatic contours were used, no such effect was found. Moreover, the use of black contours in the alternating presentation condition greatly diminished filling-in induced both by an inner and outer chromatic contour.

At first glance, the results from the alternating condition in the present experiment appear to be at odds with the results from Experiment 1. In the first experiment, an inner chromatic contour generally induced stronger afterimage filling-in as compared to an outer chromatic contour, while this did not reveal a significantly different effect in Experiment 2. A plausible cause for these apparent differential results lies in the different background luminance. More in particular, in Experiment 1, the interior area of the adapting stimulus was always isoluminant with the chromatic contour, while in this experiment, the interior area was of a different luminance. As mentioned, this was done to allow a better comparison with the typical watercolor displays, but it also caused a luminance border between the inner chromatic contour and the interior area. This luminance border, which likely remained in the afterimage (with a contrast polarity in the opposite direction), apparently prevented the colored afterimage of the chromatic contour from spreading. In contrast, color filling-in by an outer chromatic contour should be influenced less by such afterimage luminance border as this type of filling-in depends on color induction across luminance borders (Anstis et al., 1978). Indeed, comparing the plot in Figure 5 with the left plot in Figure 7, it appears that color spreading induced by the inner chromatic contour was attenuated in Experiment 2 (running additional independent *t*-tests confirmed this observation [*t*_(38)_ = 2.65, *p* < 0.05], while the strength of color spreading induced by the outer chromatic contour appears more or less the same across experiments [*p* > 0.1]). When black achromatic contours were used, the greater luminance contrast in the afterimage appeared to interfere with both effects.

Note that although afterimages of a luminance border between a contour and the adjacent area may weaken or even prevent the spreading of afterimage colors, similar luminance borders do not prevent color spreading of “real” colors in the current watercolor displays. In fact, when contours were presented simultaneously, we replicated previous findings on the watercolor illusion (Pinna et al., 2001; Devinck et al., 2005; Cao et al., 2011) and showed that an inner chromatic contour triggered same-color filling-in. Apparently, afterimage filling-in is more sensitive to luminance borders between the inducing contours and the adjacent area than filling-in triggered by “real” colors. Note that the current difference in strengths in fact deal with the induction of the filling-in, i.e., the flow from contour colors to the adjacent area. This is different from the earlier observation that, once afterimages are generated, their perceived strength strongly depends on the position of luminance contours (e.g., Daw, 1962; van Lier et al., 2009; Powell et al., 2012).

**Figure 9:**
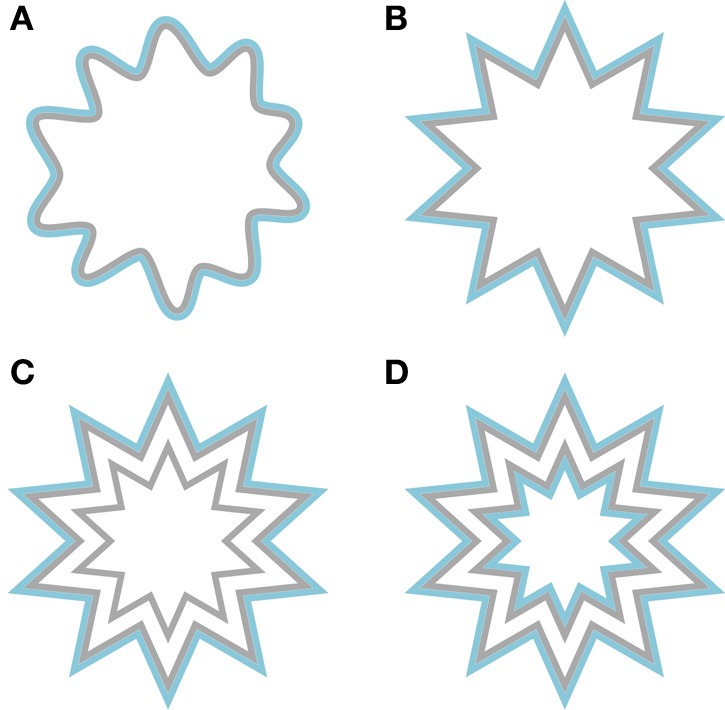
**Examples of shapes that may induce contrast induction of the blue contour. (A)** A slightly bigger version of a shape that is taken from Experiment 2. **(B)** An angular version of panel **(A)**. **(C)** Similar to panel **(B)**, but with an additional gray contour. **(D)** A jagged annulus that is flanked on both sides by a blue contour.

An additional difference between the current filling-in of afterimage colors and “real” colors appeared when filling-in was triggered by the outer chromatic contours. For afterimage filling-in, our results were in line with Experiment 1 and previous research (Anstis et al., 1978; van Lier et al., 2009). As it is likely that the effect depends on contrast induction (either of afterimage colors or of “real” colors), we also expected to find, like Pinna (2006), contrast induction when “real” colors were used. However, this is not what we found; not only was color filling-in triggered by the outer contours stronger for afterimage colors as compared to “real” colors, color filling-in triggered by “real” colors was, unexpectedly, more likely to be of the same color as the inducing contour (see Figures 7, 8, right plots). We speculate that given the very weak color appearance for these configurations, color judgments were biased toward the color of contour that was actually present in the display. The use of different stimulus configurations across studies may further offer a solution for the diverging results. To illustrate this, consider the shapes in Figure 9. In the upper left and upper right, one of the shapes used in our experiment (Figure 9A) and an angular version of that shape (Figure 9B) are shown. Both shapes produce rather weak, but an approximately similar color appearance based on contrast induction (i.e., orangish filling-in) in the interior area, indicating that the type of contour (smoothly undulating or angular) does not seem to matter much. However, the effect can be enhanced by reducing the to be filled-in area by adding a gray contour inside the inner area (Figure 9C). The enclosed area between the gray contours now reveals a stronger orangish impression. In Figure 9D we have added an additional blue contour outside that area. As a consequence the inner part of the figure has a bluish tint and the area between the gray contours appears even more orangish. In fact, the latter figure is similar to the examples provided by Pinna (2006). These informal observations illustrate that our stationary stimuli were perhaps not optimized for strong filling-in based on contrast induction. Note further that, contrary to shapes such as Figure 9D in which contrast induction can be compared with watercolor filling-in within the inner region, our stimuli were presented in isolation, which may have made the task of judging color filling-in of an already weak effect even more challenging.

## General discussion

We showed that, similarly to the watercolor illusion, afterimages of thin colored outlines spread within a region bounded by luminance contours. Spreading induced by an afterimage of an inner chromatic contour appears stronger as compared to spreading induced by an afterimage of an outer contour. The probability of perceiving filling-in depended on whether it is induced by “real” colors or afterimage colors. For instance, it appears that spreading of afterimage colors can be more easily affected by changes in luminance settings as compared to spreading of “real” colors. In addition, afterimage colors were, in contrast to “real” colors, more likely to induce color contrast across boundaries.

Filling-in demonstrated in this study may be related to other filling-in phenomena that appear to depend on mechanisms that are related to boundary processing. For example in Troxler fading, prolonged fixation causes stimuli in the periphery (Troxler, 1804), or, as has been shown more recently, even entire scenes (Simons et al., 2006) to disappear from view. As adaptation causes luminance boundaries to break down, color may spread beyond that boundary. Filling-in due to Troxler fading has been studied using “real” colors (Hamburger et al., 2005) and afterimage colors (Hamburger et al., 2012). They also found that different types of filling-in triggered by “real” colors and afterimage colors did not perfectly match. As has been mentioned in the introduction, another possible related instance of filling-in occurs in the neon color illusion. Afterimages of neon color spreading have also been reported (Shimojo et al., 2001), which have been shown to be the result of adaptating to the illusory filled-in surface. However, in our demonstrations the situation is different. As the chromatic contours in our stimuli are not likely to induce filling-in or contrast induction by themselves (a second contour is necessary), it is likely that for our stimuli, filling-in mechanisms act on the afterimage of the chromatic contours instead.

Several theories of form and surface perception account for filling-in phenomena (Komatsu, 2006). For example (Grossberg and Mingolla, 1985) proposed that visual input is processed into two parallel systems; a boundary contour system and a feature contour system. Boundary and edge information are processed in the boundary contour system, while feature information such as color and brightness are processed in the feature contour system. A perception of a surface is formed when information of both systems are combined. Color and brightness information in the feature contour system spread across surfaces and are bound by edge information in the boundary contour system. The theory has been used to explain filling-in phenomena such as the neon color effect (Grossberg and Mingolla, 1985) and also the watercolor illusion (Pinna and Grossberg, 2005). In fact, the latter authors constructed a stimulus, the so-called two-dot limiting case, to explain both similarities and differences between the neon color illusion and the watercolor illusion.

Recently Francis (2010) used a model based on the boundary contour system and the feature contour system to simulate the perceived afterimages in van Lier et al. (2009). Initially, the results from the model were consistent with the idea that boundaries block color spreading. In a second study however, some predictions of the model did not entirely match their experimental data (Kim and Francis, 2011). Particularly, in one instance they used similar star-like stimuli (see Figure 1) as used in van Lier et al. (2009), but varied the size of the test outline. Contrary to predictions of the model, their experimental results revealed that afterimage filling-in was less likely to be perceived when the test contour did not exactly match the edges of the inducing color. The results were explained by the fact that for a smaller test contour, the region that receives afterimage color signals is relatively smaller, while for larger test contour, a larger region had to be filled in. Both of these factors may have diluted color spreading. An alternative interpretation is that the alignment of a test contour with the edges of the inducing colored region is important for filling-in to occur, because of repeated activation of orientation selective neurons that are also coding for color (Friedman et al., 2003). Kim and Francis (2011) additionally found that when the test contour was larger as compared to matching contours, the probability of perceiving filling-in of an unexpected color became higher, possibly due to the fact that larger test contours included afterimages from two complementary colors. However, when the test contour was smaller than the inducing colored region, the probability of perceiving no color filling-in became higher. In addition to their explanation, this result may have been caused by the fact that while one portion of the inducing colored region fell inside the test contour, another portion fell outside the test contour. It is possible that the similarly colored afterimages induced by these outer colors negated the complementary colored afterimages induced by the inner colors.

To illustrate this, consider the examples in Movie 3. In addition to adapting figures with only one contour (on the right), we created adapting figures comprising two chromatic contours, one of which is positioned inside and the other outside the test contour (on the left). When both contours are of the same color, complementary colored filling-in induced by the inner contour and similarly colored filling-in induced by the outer contour appear to cancel each other out, so no color filling-in is perceived (compare top left with top right animation). When the color of the outer contour is changed to purple (bottom left), both inner and outer contour induce approximately the same purplish colored afterimage, leaving a stronger purplish impression as compared to when only an inner green contour is used (compare bottom left with bottom right). Another example is provided in Movie 4. Here the chromatic contour is sequentially followed by two achromatic contours, juxtaposed to the chromatic contour; one positioned inside the interior area, and one positioned outside the interior area. After viewing a few cycles (remain fixated on the central dot) one may see a color change with regard to the afterimage filling-in, corresponding with the position of the contour. These examples illustrate that both effects play an important role in afterimage filling-in and should be integrated in any model accounting for afterimage filling-in.

All in all, the current color filling-in triggered by alternating juxtaposed chromatic and achromatic contours largely reveals similar phenomenological impressions as the original watercolor illusion. Nevertheless, there are also different sensitivities for both types of filling-in. It should be noted, however that phenomenological differential effects for “real” colors and afterimage colors do not necessarily point to fundamentally different processing mechanisms between these types of color. For example, it has been shown that when having appropriate color settings (e.g., having “real” colors that are more comparable to the relatively weak and desaturated afterimage colors), both types of colors can be effectively blocked and gated by luminance contours (e.g., Anstis et al., 2012a,b). Further investigations (e.g., Powell et al., 2012) may clarify whether different sensitivities are merely a result of different stimulus parameters and color properties (like saturation) or whether they are caused by different underlying mechanisms. The current afterimage watercolors may provide a suitable entrance to further examine the conditions under which colors straddle the boundaries.

### Conflict of interest statement

The authors declare that the research was conducted in the absence of any commercial or financial relationships that could be construed as a potential conflict of interest.

## Supplementary material

Movie 1 **Examples of stimuli in which the inner contour is the chromatic adapting contour**. Observers tend to perceive color filling-in complementary to the color of the adapting contour. Note, the effect appears somewhat stronger after a couple of alternations (please set your video player to loop the movie and fixate on the small dot in the middle of the four stimuli).

Movie 2 **Examples of stimuli in which the outer contour is the chromatic adapting contour**. Observers tend to perceive color filling-in similar to the color of the adapting contour.

Movie 3 **Examples of stimuli with two chromatic adapting contours**. In the top left a stimulus is shown comprising both an inner and an outer green adapting contour. Compared with the figure in the top right, color spreading appears less strong, because filling-in induced by both contours appear to cancel each other out. In contrast, when the outer contour is changed to purple (lower left), both contours induced a similar colored afterimage. The color should be similar to color filling-in induced by the stimulus in the lower right.

Movie 4 **Examples of chromatic contours that are followed subsequently by two different achromatic contours**. After a few cycles the color of the afterimages switches according to the achromatic contour.
